# Preliminary study of coronavirus disease 2019 on pets in pandemic in Denpasar, Bali, Indonesia

**DOI:** 10.14202/vetworld.2021.2979-2983

**Published:** 2021-11-26

**Authors:** Hamong Suharsono, Ali Ghufron Mukti, Ketut Suryana, I. Wayan Masa Tenaya, Dilasdita Kartika Pradana, Guy Daly, Mochamad Panji Pujasakti

**Affiliations:** 1 Department of Biochemistry, Faculty of Veterinary, Udayana University of Denpasar, Bali, Indonesia; 2 Research and Innovation Consortium for COVID-19, Ministry of Research and Technology/National Agency of Research and Innovation, Jakarta, Indonesia; 3 Department of Internal Medicine at Wangaya Hospital in Denpasar, Bali, Indonesia; 4 Veterinary Disease Investigation Centre, Denpasar, Bali, Indonesia; 5 Coventry University, Priory St, Coventry CV1 5FB, United Kingdom; 6 International Coordinating Research, Ministry of Research and Technology/National Agency of Research and Innovation, Jakarta, Indonesia

**Keywords:** pets, severe acute respiratory syndrome coronavirus-2, viral detection

## Abstract

**Background and Aim:**

Coronavirus disease 2019 (COVID-19) is an acute infectious respiratory disease caused by severe acute respiratory syndrome coronavirus-2 (SARS-CoV-2) and has spread rapidly globally, resulting in a pandemic. In humans, the main routes of transmission are respiratory droplets and close contact with infected individuals or through contact with an object infected with the virus, followed by touching mouth, nose, or eyes. It is assumed that SARS-CoV-2 was originated in wild animals and was then transmitted to humans. Although some wildlife and domestic animals can be naturally or experimentally infected with the virus, the intermediate hosts that transmitted it to humans are still unknown. Understanding the dynamics of SARS-CoV-2 associated with possible zoonotic transmission of intermediate hosts is considered critical. Reportedly, cats or dogs living with COVID-19-positive humans tested positive for the disease, suggesting that the virus was transmitted to the animals from humans. Information regarding the epidemiological investigation and comprehensive studies is limited. Therefore, it is still unclear how high is the correlation of infection in humans and pet animals, especially those living together. The aim of this study was to investigate the possibility of SARS-CoV-2 infection in the pets of patients with COVID-19 who were hospitalized at the Wangaya hospital, Denpasar, Bali, Indonesia.

**Materials and Methods:**

A total of seven clinically asymptomatic pets (six dogs of different races and sexes and a cat [age, 360-2920 days]) were included in this study. These animals belonged to patients with confirmed SARS-CoV-2 infection from August to November 2020. Nasal swab and nasopharyngeal samples were collected from the pets individually under anesthetic condition and were collected 6-12 days after confirmed SARS-CoV-2 infection in owners and hospitalization at the Wangaya Hospital. The swab samples were then processed for RNA isolation and tested using reverse transcription-polymerase chain reaction (RT-PCR) for SARS-CoV-2, in accordance with the World Health Organization manual 2020.

**Results:**

RT-PCR results for all seven RNA samples, prepared from the swab samples, were negative. For the samples, all PCR products were below the threshold limit, suggesting no genetic material belonging to the samples tested.

**Conclusion:**

This was the first preliminary study of COVID-19 on pets in pandemic using RT-PCR. The study tested a very limited quantity of samples, and all of them were negative. However, the way in which the samples were prepared was considered appropriate. Therefore, in further studies, testing of more samples of pets of more individuals with confirmed SARS-CoV-2 infection is required.

## Introduction

Severe acute respiratory syndrome coronavirus-2 (SARS-CoV-2) originated in Wuhan, China, in December 2019. On February 17, 2020, the World Health Organization (WHO) reported that SARS-CoV-2 had spread globally [1,2]. The incubation period for SARS-CoV-2 is 1-14 days, mostly 3-7 days, and the main routes of transmission are respiratory droplets and close individual-to-individual contact (within 1 m). SARS-CoV-2 mainly infects the lungs in the early stages and probably attacks, to a lesser degree, other organs that express angiotensin-converting enzyme II, such as the gastrointestinal system and the kidneys. Respiratory dysfunction and failure are the major threats to patients as well as the major cause of death [3]. One of the most significant and severe clinical signs occurring in humans is a respiratory symptom, as the virus targets the lungs; the infection is also sometimes accompanied by other clinical symptoms such as fever and diarrhea. Transmission might occur if an individual touches an object infected with the virus and then touches mouth, nose, or eyes [4-7]. Presumably, SARS-CoV-2 originated from wild animals and was then transmitted to humans. Although some wildlife and domestic animals, including cats and dogs, can be infected with the virus, the intermediate hosts that transmitted the virus to humans are still unknown. Reportedly, cats and dogs have been reported to be susceptible to alphacoronaviruses and betacoronavirus, known as feline CoVs and canine CoVs [8,9]; therefore, these animals may also be susceptible to betacoronavirus.

Understanding the dynamics of SARS-CoV-2 associated with the possibility of zoonotic transmission of intermediate hosts is considered critical. Reportedly, cats or dogs living with ­SARS-CoV-2-infected humans tested positive, suggesting that animals go the infection from humans. However, information regarding clinical aspects and comprehensive studies are still limited. A high frequency of close contact between infected individuals and their pets is considered to be a critical factor in transmitting the virus from humans to pets. The WHO suggests that the present spread of SARS-CoV-2 is an aftereffect of individual-to-individual transmission; therefore, SARS-CoV-2-infected patients should restrict contact with other individuals, pets, and all kinds of animals. Infected humans should maintain adequate hand hygiene practice, including washing hands before interacting with pets and wearing facemasks in public places [6,7,10,11].

To understand the dynamics of SARS-CoV-2 associated with the possibility of zoonotic transmission of intermediate hosts, this study investigated the possibility of SARS-CoV-2 infection in pets belonging to owners with coronavirus disease 2019 (COVID-19) who were hospitalized at the Wangaya Hospital, Denpasar, Bali, Indonesia.

## Materials and Methods

### Ethical approval and Informed consent

Institutional review board approval for this study was obtained from the Ethical Committee of Research of Wangaya Hospital (reference number: 09/RSUDW/Litbang/2020). The written consent was obtained from the owners before the commencement of the study.

### Study period and location

The study was conducted from August to November 2020. A total of seven asymptomatic pets (six dogs of different races and sex and one cat; age, 360-2920 days) were used in this study. The samples were collected between 6 to 12 days of confirmed SARS-CoV-2 infection in pet owners and hospitalization at the Wangaya Hospital in Denpasar, Bali, Indonesia (Table-1). Two samples per animal (nasal and nasopharyngeal swabs) were collected. All selected pets belonged to individuals who had tested positive for COVID-19 and had close daily interaction with pets, as they were considered as family members. The suspected pets belonged to six individual pet owners who had confirmed SARS-CoV-2 infection from August to November 2020 and were hospitalized at the Wangaya Hospital in Denpasar, Bali, Indonesia.

**Table-1:** Characteristics of pets screened for SARS-CoV-2 living with owners who confirmed positive with COVID-19.

Sample (COVID-19)	Pets (species)	Breed	Age (days)	Sex	COVID-19 disease severity in pet owners	Days from owner’s COVID-19 confirmed to pet sample collection
1	Dog	Mix Shitsu	2920	Male	Non-severe (fever, dry cough, anorexia, respiratory rate 24 [breath/min], SpO_2_: 94% in resting state)	6
2	Dog	Mix Kintamani	730	Male	Non-severe (fever, dry cough, headache, respiratory rate 22 [breath/min], SpO_2_: 95% in resting state)	9
3	Dog	Mix Pom	730	Female	Severe (fever, dry cough, anosmia, nausea, anorexia, shortness of breath; respiratory rate 34 [breath/min], SpO_2_: 92% in resting state)	12
4	Dog	Mix Shitsu	1460	Male	Severe (fever, dry cough, anosmia, nausea, vomiting, shortness of breath; respiratory rate 36 [breath/min], SpO_2_: 90% in resting state)	12
5	Dog	Mix Pom	360	Male	Non-severe (fever, dry cough, myalgia, respiratory rate 24 [breath/min], SpO_2_: 95% in resting state)	8
6	Cat	Local	360	Female	Non-severe (fever, dry cough, anorexia, respiratory rate 22 [breath/min], SpO_2_: 96% in resting state)	6
7	Dog	Mix Kintamani	360	Male	Non-severe (fever, dry cough, headache, respiratory rate 24 [breath/min], SpO_2_: 95% in resting state)	7

COVID=Coronavirus disease, SARS-CoV-2=Severe acute respiratory syndrome-coronavirus-2

### Methods

The severity of SARS-CoV-2 in humans was classified as follows: Severe cases and critical cases. The critical cases met one of the following criteria: Respiratory failure and requiring mechanical ventilation or shock, or other organ damage combined with hospitalization in the intensive care unit. The samples were collected and tested for SARS-CoV-2 using protocols provided in the WHO 2020 manual [12], with slight modification. First, biosafety and biosecurity considerations were strongly implemented to prevent disease transmission from animals to officers and/or to the environment. This was primarily done by washing hands with soap/disinfectant and using personal protective equipment. For safety reasons associated with animal welfare practices, targeted pets were anesthetized before samples collection from nasal and oral cavities. The swab samples were obtained using a plastic stem from the upper respiratory tract (nasopharyngeal swab) and oropharynx. Then, the samples were properly placed in sterile tubes containing viral transport media and then sent to the Biotechnology Section of Disease Investigation Center in Denpasar for further analysis. Targeted viral RNAs were then isolated from the swab samples according to the QIAamp viral RNA Mini Kit (Qiagen,-USA), before being amplified by the RdRP gene using One-Step RT-AgPath-ID One-Step reverse transcription-polymerase chain reaction (RT-PCR)^®^ (Applied Biosystems, Foster City, CA, USA). The RT-PCR master mix was 25 μL and comprised 12.5 μL 1×RT-PCR reaction buffer, 1 μL of forward primer, 1 μL of reverse primer, 1 μL of specific probe (concentration: 5 pmol/μL), 3.5 μL of dH2O, 1 μL of RT-PCR enzyme mix, and 5 μL of template. cDNA synthesis was conducted by reverse transcription method at 55°C for 5 min. PCR steps included 1 cycle of pre-denaturation at 95°C for 5 s followed by an amplification of 40 cycles with each of the following temperatures: Denaturation of 95°C for 5 s, annealing 60°C for 60 s, and extension at 68°C for 15 s.

## Results

**Figure-1:**
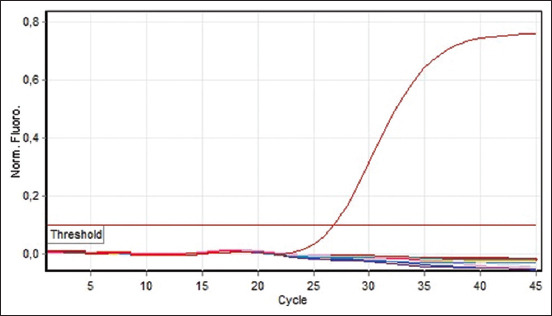
Reverse transcription-polymerase chain reaction of RNA samples from coronavirus disease 2019-suspected pets. Positive control reaction (arrow).

RT-PCR used in this study was specific and sensitive, with the control positive RNA showing consistent positive results (Figure-1). In contrast, no positive results were detected in the negative control. Furthermore, all seven RNA samples obtained from seven pets were PCR negative; that is, results of all RT-PCR reactions were below the threshold limit (Figure-1).

Five owners were noted to have suffered from non-severe COVID-19 and one had severe COVID-19. Samples were collected at various time points from confirmation of COVID-19 in owners, ranging from 6 to 12 days (Table-2).

**Table-2:** SARS-CoV-2 RT-PCR results from seven individual the pets tested.

Sample (COVID-19)	Pets (species)	Breed	Age (days)	Gender	SARS-CoV-2 RT-PCR results		COVID-19 disease severity in pet owners	Days from owner’s COVID-19 confirmed to pet sample collection

					Nasal swab	Oropharyngeal swab		
1	Dog	Mix Shih Tzu	2920	Male	Negative	Negative	Non-severe	6
2	Dog	Mix Kintamani	730	Male	Negative	Negative	Non-severe	9
3	Dog	Mix Pom	730	Female	Negative	Negative	Severe	12
4	Dog	Mix Shih Tzu	1460	Male	Negative	Negative	Severe	12
5	Dog	Mix Pom	360	Male	Negative	Negative	Non-severe	8
6	Cat	Local	360	Female	Negative	Negative	Non-severe	6
7	Dog	Mix Kintamani	360	Male	Negative	Negative	Non-severe	7

COVID = Coronavirus disease, SARS-CoV-2 = Severe acute respiratory syndrome-coronavirus-2, RT-PCR = Real time-polymerase chain reaction

## Discussion

There is still limited evidence confirming that SARS-CoV-2 spreads by pets and other animal companions to individuals. However, the WHO and Centers of Disease Control have consistently recommended that individuals limit their contact with their pets, probably to reduce the risk of infecting their pets with the virus, in whom the virus can replicate. Accordingly, the present study was conducted to provide further confirmation regarding whether pets in close contact with individuals with confirmed COVID-19 harbor or do not harbor the virus. We found that all RNA samples prepared from the seven suspected pets, tested using RT-PCR, and were negative; this was irrespective of the fact that the pets belonged to owners with a history of SARS-CoV-2 positivity. Furthermore, house interaction (daily close contact) was prevalent between the owners and their pets as well as with other family members. As the RNA samples tested were not positive through PCR, the current findings suggested that no viral gene associated with SARS-CoV-2 was found in the animals tested. This result possibly supports findings reported to date [6,10,13]. Consequently, pets may not be susceptible to COVID-19, unlike humans. However, further research may be required to ­confirm the current findings and to determine transmission from pets to humans. This is because the findings may also be related to the limited number of pets enrolled in our study, conferring a bias in the results. However, limited studies have been conducted showing that pets, such as dogs and cats, can be infected by COVID-19 [11,13-19]. In March 2020, Parry reported that a 30-year-old woman in Hong Kong was diagnosed with COVID-19 after her dog tested positive for the virus. In Belgium, cat tested positive for SARS-CoV-2 1 week after its owner was diagnosed with COVID-19 [1]. If pets develop an unexplained illness and have been exposed to an individual with SARS-CoV-2, their owners should inform public health officials or public health veterinarians who will advise the owner to take the pet to a veterinary clinic. There is a lack of evidence supporting the view that pets have a role in the epidemiology of SARS-CoV-2. Even so, the entire clinical team should maintain a good level of hygiene practice throughout the veterinary interaction, particularly with animals in contact with infected individuals. Pet owners, themselves, should limit their contact with their pets and always wash hands before and after interacting with their pets [20-23].

## Conclusion

This study assessed the presence of SARS-CoV-2 infection in pets (dogs and cats) living in close contact with owners in Bali, although with a limited sample (six dogs and one cat). All RT-PCR results from pet specimens (nasal and oropharyngeal swabs) were negative. Therefore, a further study will be conducted for testing more pets with PCR using SARS-CoV-2 primers. This information will be critical in taking a collaborative transdisciplinary One Health approach to help prevent future SARS-CoV-2 outbreaks.

## Authors’ Contributions

HS: Made substantial contribution in conception and design, acquisition of data and analysis, drafted and revised the manuscript for ensuring critical academic content. AGM, IWMT, GD, and MPP: Drafted and revised the manuscript for ensuring critical academic content. KS and DKP: Participated in collecting data and drafting and reviewing the manuscript, and revising it for ensuring critical academic content. All authors read and approved the final manuscript.

## References

[1] Parry N (2020). COVID-19 and pets:When pandemic meets panic. Forensic Sci. Int. Rep.

[2] Kolifarhood G, Aghaali M, Saadati H.M, Taherpour N, Rahimi S, Izadi N, Nazari S.S (2020). Epidemiological and clinical aspects of SARS-COV-2;a narrative review. Arch. Acad. Emerg. Med.

[3] Yi Y, Lagniton P.N.P, Ye S, Li E, Xu R.H (2020). SARS-COV-2:what has been learned and to be learned about the novel coronavirus disease. Int. J. Biol. Sci.

[4] Abdulamir A.S, Hafids R.R (2020). The possible immunological pathways for the variable immunopathogenesis of SARS-COV-2 infection among healthy adults, elderly, and children. Electron. J. Gen. Med.

[5] Islam M.A (2020). SARS-COV-2 and pet animals:What we need to know?. Res. Agric. Fish.

[6] Patterson E.I, Elia G, Grassi A, Giordano A, Desario C, Medardo M, Smith S.L, Anderson E.R, Prince T, Patterson G.T, Lorusso E, Lucente M.S, Lanave G, Lauzi S, Bonfanti U, Stranieri A, Martella V, Basano F.S, Barrs V.R, Radford A.D, Agrimi U, Hughes G.L, Paltrinieri S, Decaro N (2020). Evidence of exposure to in cats and dogs from households in Italy. Nat. Commun.

[7] World Organization for Animal Health Infection with in Animals.

[8] Le Poder S (2011). Feline and canine coronaviruses:Common genetic and pathological features. Adv. Virol.

[9] Fritza M, Rosolenb B, Krafftc E, Becquarta P, Elgueroa E, Vratskikhd O, Denollye S, Bosone B, Vanhomwegendf J, Ar Gouilhgh M, Kodjoc A, Chirouzebi C, Rosolenjk S.G, Legrosce V, Leroy E.M (2020). High prevalence of antibodies in pets from SARS-COV-2+households. One Health.

[10] Contini C, Nuzzo M.D, Barp N, Bonazza A, Giorgio R.D, Tognon M, Rubino S (2020). The novel zoonotic SARS-COV-2 pandemic:An expected global health concern. J. Infect. Ctries.

[11] Decaro N, Martella V, Saif L.J, Buonavoglia C (2020). SARS-COV-2 from veterinary and one health perspectives:What animal coronaviruses have taught us. Res. Vet. Sci.

[12] World Health Organization. Diagnostic Test for SAR-Cov-2 (2020). Interim Guidance.

[13] World Health Organization (2020). Considerations in Adjusting Public Health and Social Measures in the Context of SARS-COV-2 Interim Guidance.

[14] Shi J, Wen Z, Zhong G, Yang H, Wang Z, Huang B, Liu R, He X, Shuai L, Sun Z, Zhao Y, Liu P, Liang L, Cui P, Wang J, Zhang X, Guan Y, Tan W, Wu G, Chen H, Bu Z (2020). Susceptibility of ferrets, cats, dogs, and other domesticated animals to SARS-coronavirus 2. Science.

[15] Mackenzie J.S, Smith D.W (2020). SARS-COV-2:A novel zoonotic disease caused by coronavirus from China:What we know and what we do not. Microbiol. Aust.

[16] Pan American Health Organization (2021). SARS-COV-2 Joint Announcement on the Coronavirus and Dogs and Cats.

[17] Kiros M, Andualem H, Kiros T, Hailemichael W, Getu S, Geteneh A, Alemu D, Abegaz W.E (2020). SARS-COV-2 pandemic:Current knowledge about the role of pets other animals in disease transmission. Virol. J.

[18] Applebaum J.W, Tomlinson C.A, Matijczak A, McDonald S.E, Zsembik B.A (2020). The concerns, difficulties, and stressors of caring for pets during SARS-COV-2:From a large survey of US pet owners. Animals (Basel).

[19] McNamara T, Richt J.A, Glickman L (2020). A critical needs assessment for research in companion animals and livestock following the pandemic of SARS-COV-2 humans. Vector Borne Zoonotic Dis.

[20] World Health Organization (2019). Novel Coronavirus (2019-nCoV) Situation Report-22.

[21] Hunjan U.G, Reddy J (2020). Why companion animals are beneficial during SARS pandemic. J. Patient Exp.

[22] Tiwari R, Dhama K, Sharun K, Yatoo M.I, Malik Y.S, Singh R, Michalak I, Sah R, Bonilla-Aldana D.K, Rodriguez-Morales A.J (2020). SARS-COV-2:Animals, veterinary zoonotic links. Vet. Q.

[23] Kumar D, Malviya R, Sharma P.K (2020). Corona virus:A review of SARS-COV-2. Eur. J. Med. Oncol.

